# An Evidence for a Novel Antiviral Mechanism: Modulating Effects of Arg-Glc Maillard Reaction Products on the Phase Transition of Multilamellar Vesicles

**DOI:** 10.3389/fcell.2020.629775

**Published:** 2021-01-28

**Authors:** Lijing Ke, Sihao Luo, Pingfan Rao, Jeremy P. Bradshaw, Farid Sa'adedin, Michael Rappolt, Jianwu Zhou

**Affiliations:** ^1^Food Nutrition Sciences Centre, Zhejiang Gongshang University, Hangzhou, China; ^2^Royal (Dick) School of Veterinary Studies, College of Medicine & Veterinary Medicine (MVM), The University of Edinburgh, Edinburgh, United Kingdom; ^3^School of Food Science and Nutrition, University of Leeds, Leeds, United Kingdom

**Keywords:** Maillard reaction products, phase transition, multilamellar vesicles, MeDOPE, x-ray scattering

## Abstract

Maillard reaction products (MRPs) of protein, amino acids, and reducing sugars from many foods and aqueous extracts of herbs are found to have various bioactivities, including antiviral effects. A hypothesis was proposed that their antiviral activity is due to the interaction with the cellular membrane. Aiming to estimate the possible actions of MRPs on phospholipid bilayers, the Arg-Glc MRPs were prepared by boiling the pre-mixed solution of arginine and glucose for 60 min at 100°C and then examined at a series of concentrations for their effects on the phase transition of MeDOPE multilamellar vesicles (MLVs), for the first time, by using differential scanning calorimetry (DSC) and temperature-resolved small-angle X-ray scattering (SAXS). Arg-Glc MRPs inhibited the lamellar gel–liquid crystal (*L*_β_-*L*_α_), lamellar liquid crystal–cubic (*L*_α_-*Q*_II_), and lamellar liquid crystal–inverted hexagonal (*L*_α_-*H*_II_) phase transitions at low concentration (molar ratio of lipid vs. MRPs was 100:1 or 100:2), but promoted all three transitions at medium concentration (100:5). At high concentration (10:1), the MRPs exhibited inhibitory effect again. The fusion peptide from simian immunodeficiency virus (SIV) induces membrane fusion by promoting the formation of a non-lamellar phase, e.g., cubic (*Q*_II_) phase, and inhibiting the transition to *H*_II_. Arg-Glc MRPs, at low concentration, stabilized the lamellar structure of SIV peptide containing lipid bilayers, but facilitated the formation of non-lamellar phases at medium concentration (100:5). The concentration-dependent activity of MRPs upon lipid phase transition indiciates a potential role in modulating some membrane-related biological events, e.g., viral membrane fusion.

## Introduction

The Maillard reaction is a non-enzymatic browning chemistry reaction between amino acids (or peptides, or proteins) and a reducing sugar, usually requiring heat. As a major chemical change that occurs during food processing, herb decocting, and physiological aging, Maillard reaction products (MRPs) have been associated with a number of functions and bioactivities, such as flavoring, coloring, modification of proteins and lipids with glycation, and formation of antioxidant or mutagenic compounds. Both positive and negative influences of MRPs on cell reproduction have been reported (Einarsson et al., 1983; Harris and Tan, 1999; Kundinger, 2004; Rufián-Henares and Morales, 2006). MRPs from amino acids and glucose showed significant impacts on the growth of the microorganisms (Harris and Tan, 1999). This impact varies according to which amino acid was used. MRPs derived from reaction of arginine, glycine, and histidine with glucose promoted the growth of *Staphylococcus aureus* and *Salmonella enteritidis*, while MRPs of cysteine and glucose inhibited the growth of both germs.

Maillard reaction occurs widely during the preparation of boiling water extracts of herbs including herbal traditional Chinese medicine (TCM). The decoction of botanically distinguished herbs, e.g., *Isatidis Radix, Momordica charantia*, and ginseng, possesses antiviral activities by inhibiting influenza A virus adsorption on epithelial cells (Chen et al., 2006; Ke et al., 2012). Arginine and glucose are the most abundant Maillard reactants found in these herbs, implying that arginine-glucose MRPs (Arg-Glc MRPs) are the representative of MRPs in the decoction. Hemagglutination is mediated by the binding of viral envelope glycoprotein hemagglutinin (HA) to cellular plasma membrane receptors, sialic acid residues of glycolipids (Rogers et al., 1985; Wiley and Skehel, 1987; Kobasa et al., 2004). Arg-Glc MRPs inhibited the attachment of influenza virus to erythrocytes, which indicates that they may tackle the interaction between HA and lipid membrane of cells (Ke, 2010).

In order to infect the host cell, both enveloped and non-enveloped viruses have to penetrate the barrier of a cellular membrane. For enveloped viruses, influenza virus A for example, penetration involves membrane fusion. For non-enveloped viruses, picornaviruses, for instance, penetration involves membrane lysis or pore formation (Marsh and Helenius, 2006). Non-lamellar structures have been discovered either at the sites of membrane fusion or membrane pore formation. Peptides or proteins, which promote membrane fusion or lyse membrane, facilitate the formation of non-lamellar phases, either micelles, cubic phases, or hexagonal phases (Epand, 1998). Membrane fusion is a critical early event for an influenza virus to transfer its genetic information to human epithelial cells and complete its replication. From there, the new virus particles are formed and released to infect other cells. Nevertheless, membrane fusion is also involved in the budding of newly formed virus particles before they are released from the host cells.

In this study, we set off to elucidate whether Arg-Glc MRPs interrupt viral infection by interacting with lipid bilayers of the cells and blocking the formation of non-lamellar phases, hiring the MLVs of an unsaturated phosphatidylethonalamine as model vesicles. The potential inhibitory effects of MRPs on peptide-induced membrane fusion were examined, using simian immunodeficiency virus (SIV) fusion peptide as an example.

## Methods and Materials

### Materials

1,2-Dioleoyl-sn-glycero-3-phosphoethanolamine-N-methyl (MeDOPE) was purchased from Avanti Polar Lipids Inc., USA, and used without further purification. Chloroform, methanol, and buffers are all graded AR and purchased from Sigma-Aldrich (Irvine, UK).

### Sample Preparation

For X-ray diffraction measurement, the MLVs were prepared by dispersing 15 wt% of MeDOPE in PIPES buffer [pH 7.4, 20 mM Piperazine-N,N'-bis(2-ethanesulfonic acid), 150 mM NaCl], Sigma-Aldrich (Irvine, UK). Various samples including Arg-Glc MRPs and SIV fusion peptide (GVFVLGFLGFLA, >99%) were dissolved in deionized water or methanol and mixed thoroughly with lipid MLVs by vigorous vortex for 2 min.

### Differential Scanning Calorimetry

MeDOPE MLVs were prepared as reported previously (Harroun et al., 2003) with modifications. After the lipids were dissolved in chloroform, the samples were added and mixed thoroughly by vortex and sonication. The lipid/sample suspensions were then dried up with nitrogen stream and left in vacuum overnight. Lipid concentration in the corresponding vesicle suspension was 100 mM for all the samples. MRPs were dissolved in PIPES buffer (pH 7.4, 20 mM, 150 mM NaCl) added as a serial molar ratio of 0, 1, 5, 10, 20, 30, 40, 50–100 lipid molecules. The SIV peptide was dissolved in methanol and added to the lipid solution at 1 and 2 mol%.

The dried lipid film was rehydrated with PIPES buffer at a temperature higher than *T*_M_ and vortexed to make 100 mM vesicle suspension. The suspension was sonicated for 1–2 min and soaked in liquid nitrogen (−180°C). The suspension was then defrosted at a temperature of at least 20°C above the *T*_M_. The freeze–thaw cycle was repeated six times to obtain a unified lipid packing by wiping off the memory of lipids on their thermal history. The vesicles were mixed thoroughly prior to being injected and sealed into the aluminum sample pan. The pans were weighted as both empty and sealed; thereby, the actual amount of lipids sealed in the pan was calculated. A Pyris 1 Differential Scanning Calorimeter (Perkin Elmer, USA) was employed, at a scan rate of 40°C/min. The sample chamber held 30 μl of vesicle suspension. Continuous heating scans were run from −30°C through to 85°C for MeDOPE MLVs (Sykora et al., 2005).

The transition peak was analyzed with the curve-fitting program (Pyris) based on non-linear least-squares minimization. The onset phase transition temperature (*T*_M_*, T*_Q_, and *T*_H_ for melting transition, transition to cubic phase, and transition to hexagonal phase, respectively), energy consumption (Δ*H*_f_), transition peak height (*h*), and peak area (*A*) were calculated automatically by the software. The transition peak height and peak area were then used to calculate the transition temperature range Δ*T* (Equation 1), presenting the homogeneous degree of phases existing in the phase transition.

(1) $$\begin{array}{r} \text{Δ}T=\frac{2\times A\times T}{h\times t} \end{array}$$

T, temperature from the scan rate (°C); t, time from the scan rate (s).

### Temperature-Resolved SAXS

The X-ray diffraction experiments were performed on the Austrian SAXS beamline at ELETTRA, Trieste, Italy (Amenitsch et al., 1998; Rappolt et al., 2003). Diffraction patterns of MeDOPE MLVs were recorded by a one-dimensional position-sensitive detector (Petrascu et al., 1998) covering the corresponding *s*-range of interest from ~1/450 to 1/12 Å^−1^ [*s* = 2πSin(θ)/λ]. As shown in **Figures 4**, **5**, the angular calibration was performed with silver-behenate [CH_3_(CH_2_)_20_ -COOAg] for the detector: *d001* = 58.378 Å, λ = 1.54 Å (Huang et al., 1993). The specimen-to-detector length was ~0.75 m. Equation (2) was obtained for the calculation of *s*-range by drawing a linear curve of “*s*” as a function of detector channels. Each sample was sealed in a steel chamber with a pair of thin mica film on both the entrance and exit windows, held in a steel block that was in thermal contact with a water circuit connected to a programmable temperature control unit (Unistat CC, Huber, Offenburg, Germany). The temperature was continuously monitored with a thermocouple fixed to the sample chamber in a linear fashion at a heating rate of 60 K/h, written into the data files automatically. Each frame of data collection lasted for 10–20 s depending on the scattering intensity, and for every 0.5°C (collecting for 10 s) or 1.0°C (collecting for 20 s). The X-ray beams conduct a minimal effect of thermal radiation.

### X-Ray Diffraction Data Analysis

The raw data were corrected for detector efficiency. The background scattering of water and the sample chamber was subtracted from the corrected raw data. The location, width, and amplitude of each Bragg peak were then fitted by Lorentzian distributions (SigmaPlot, Systat Software Inc.). After the sample temperature curve was drawn as a function of frames, the transition temperature of each sample was determined by identifying the initiate point of non-lamellar phases. The square root of the peak intensity was used for determination of the form factor *F* of each individual reflection. The electron density maps of the phospholipid samples in the *H*_II_ phases were derived from the small-angle x-ray diffractograms by standard procedures (e.g., see Harper et al., 2001; Rappolt et al., 2003).

The following equation was used for calculating the *s*-range of diffraction.

(2) $$S({\text{Å}}^{-\text{1}})=0.0001\text{×channel−}0.008,R^{2}=1.000$$

## Results

Lipids with smaller head groups and bigger tail groups present a cone shape. This type of lipids, e.g., MeDOPE, forms non-lamellar phases and allows us to monitor the influence of MRPs on the lamellar to non-lamellar phase transition. Primarily, the MeDOPE forms three types of structure in its aqueous dispersions: lamellar, cubic, and hexagonal, depending on the temperature, concentration, and thermal history. The non-lamellar structures are believed to relate to the initiation of peptide-induced membrane fusion. The presence of non-lamellar structure in MeDOPE samples indicates a destabilization of the lipid layers; thus, MeDOPE MLVs were used as a model lipid system for evaluating the effects of MRPs on membrane fusion induced by the SIV fusion peptide.

### Arg-Glc MRPs Affected Phase Transitions of MeDOPE MLVs

**Figure 1:**
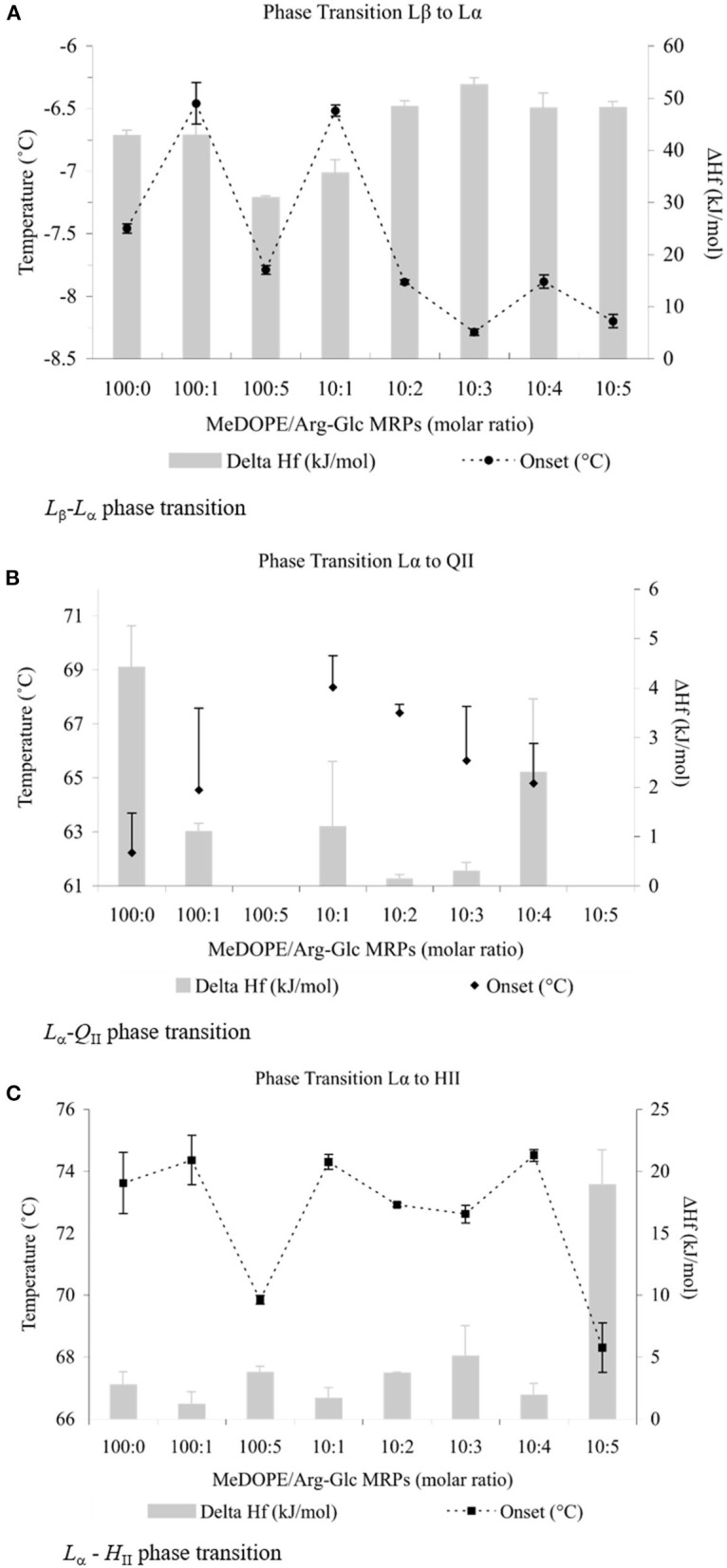
Thermodynamic effects of Arg-Glc MRPs on lipid phase transitions of MeDOPE MLVs. Examined by differential scanning calorimeter at the scan rate of 40 K/min. The continuous heating scans were performed from −30 to 85°C and repeated for at least five times for each sample. **(A)** Lamellar–lamellar (*L*_β_-*L*_α_) phase transtition. 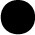, *T*_M_; 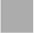, Δ*H*_f_ (*n* = 4; *P* < 0.01). **(B)** Lamellar–inverted cubic (*L*_α_-*Q*_II_) phase transition. 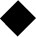, *T*_Q_; 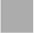, Δ*H*_f_ (*n* = 4; *P* < 0.05). **(C)** Lamellar–inverted hexagonal (*L*_α_-*H*_II_) phase transition. 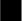, *T*_H_; 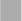, Δ*H*_f_ (*n* = 4; *P* < 0.01).

Three phase transitions of MeDOPE MLVs, *L*_β_-*L*_α_, *L*_α_-*Q*_II_, and *L*_α_-*H*_II_, were observed by DSC. In general, as shown in Figure 1, Arg-Glc MRPs exhibited the concentration-dependent effects on the phase transitions. Despite the transition temperatures of all three phases being fluctuated with MRP concentration, MRPs increased the transition temperatures at moderate concentrations (lipid:MRPs = 10:1 by molar ratio), but decreased them at the higher concentrations, particularly at the highest concentration of 10:5 by molar ratio. The influences of Arg-Glc MRPs on Δ*H*_f_ varied among the three phase transitions. When the *L*_β_-*L*_α_ transition reported the highest heat consumption of 30–50 kJ/mol, the *L*_α_-*Q*_II_ reported the least, which was generally below 2 kJ/mol. The heat consumption of the *L*_α_-*H*_II_ transition was higher than that of *L*_α_-*Q*_II_, around 5 kJ/mol and below.

As shown in Figure 1A, the initial *L*_β_-*L*_α_ phase transition temperature (*T*_M_) was ~−7.5°C, consistent with a previous report (Kusube et al., 2006). Arg-Glc MRPs decreased this temperature gradually to ~−8.2°C along the increasing concentration, with an exceptional but significant increase of 1.0°C at the molar ratio 10:1. Although both the decrease and increase were small by numbers, they were statistically highly significant (*n* = 4; *P* < 0.01). The presence of 1 mol% MRPs did not affect the *T*_M_ significantly, but remarkably increased the standard errors of measurements. The Δ*H*_f_ was decreased to ~30 kJ/mol at 5 mol% of MRPs and then gradually increased along the increasing concentrations of MRPs and stabilized at ~50 kJ/mol.

The MRPs increased the initial temperature of transition (*T*_Q_) significantly from 62 to 68°C at the molar ratio of 10:1 (as shown in Figure 1B). At either lower or higher ratios, MRPs decreased the *T*_Q_ with greater standard errors (*P* < 0.05). No *L*_α_-*Q*_II_ phase transition was observed at molar ratios of 100:5 or 10:5. The Δ*H*_f_ was decreased from 4 to ~1 kJ/mol in the presence of 1 mol% Arg-Glc MRPs.

In comparison, Arg-Glc MRPs possessed little influence on the *L*_α_-*H*_II_ phase transition with two exceptions (as shown in Figure 1C). The first, Arg-Glc MRPs (5 mol%) decreased the *L*_α_-*H*_II_ phase transition temperature (*T*_H_) from 73.6°C of pure MeDOPE to 69.9°C, and to 68.3°C at the higher molar ratio of lipid:MRPs (10:5). The second, the Δ*H*_f_ was dramatically increased to 19 kJ/mol at the molar ratio of 10:5.

SIV peptide slightly decreased the *T*_M_ of MeDOPE MLVs (*P* < 0.05 at 100:2) but increased *T*_H_ by 4°C (*P* < 0.01). In terms of transition temperatures and heat consumptions, the higher SIV concentration (100:2) was eventually not more effective than 100:1, although it did result in the greater standard errors. The heat consumptions of both transitions were reduced by SIV (*P* < 0.01). The peptide decreased the temperature range of lamellar/non-lamellar phase transition by 2°C, but slightly increased that of gel-liquid crystalline phase transition by about 0.3°C (*P* < 0.01 for 100:1 SIV).

### Arg-Glc MRPs Affected MLV Phase Transitions in the Presence of SIV Fusion Peptide

As reported previously, the SIV fusion peptide induces membrane fusion by oblique insertion into the phospholipid bilayers, promoting negative curvature, and thereby encouraging the formation of non-lamellar structures of lipid packing around the insertion site.

**Figure 2:**
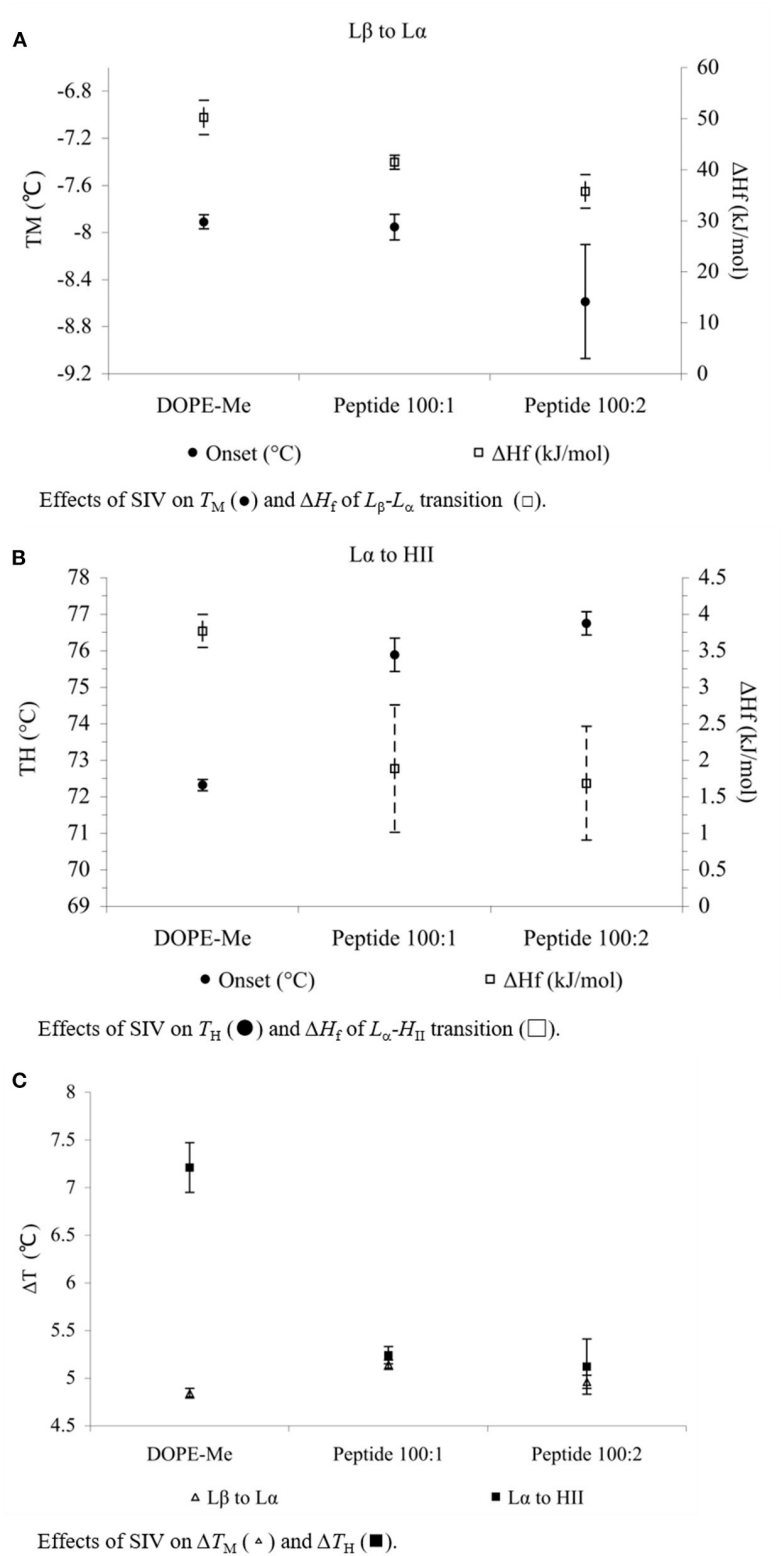
Effects of SIV peptide on phase behavior obtained in MeDOPE MLVs with DSC. Scan rate: 40 K/min. Temperature range of scanning: −30 to 85°C. Lipid concentration: 100 mM (*n* = 5; *P* < 0.01).

The influences of Arg-Glc MRPs on the peptide-induced membrane fusion were observed by determination of the phase transition profiles of MeDOPE MLVs in the presence or absence of SIV fusion peptide. In the *L*_β_-*L*_α_ phase transition (as shown in Figure 2A), the *T*_M_ of MeDOPE vesicles was −7.5 ± 0.05°C, which was decreased by SIV peptide to −8.0°C. The MRPs increased the *T*_M_ of containing 100:1 SIV peptide by 0.8°C at 100:2 (*P* < 0.01), decreased the *T*_M_ to −8.2°C at 100:5 (*P* < 0.05) and increased *T*_M_ back to −7.5°C at 10:1 (*P* < 0.01). SIV peptide decreased the temperature range (Δ*T*) of this phase transition in MeDOPE vesicles to 5.1°C (as shown in Figure 2C) when Arg-Glc MRPs increased it to 5.7°C at 10:1 (*P* < 0.01). However, in the presence of 100:1 SIV, the MRPs increased the temperature range of *L*_β_-*L*_α_ phase transition by 0.4°C at 100:5 (*P* < 0.05) and then decreased it by 0.3°C at 10:1 (*P* < 0.01). MRPs promoted the transition at 100:5 (lipid/MRPs molar ratio) but inhibited the transition at the lower (100:2) or higher concentrations (10:1) in the presence or absence of SIV peptide.

**Figure 3:**
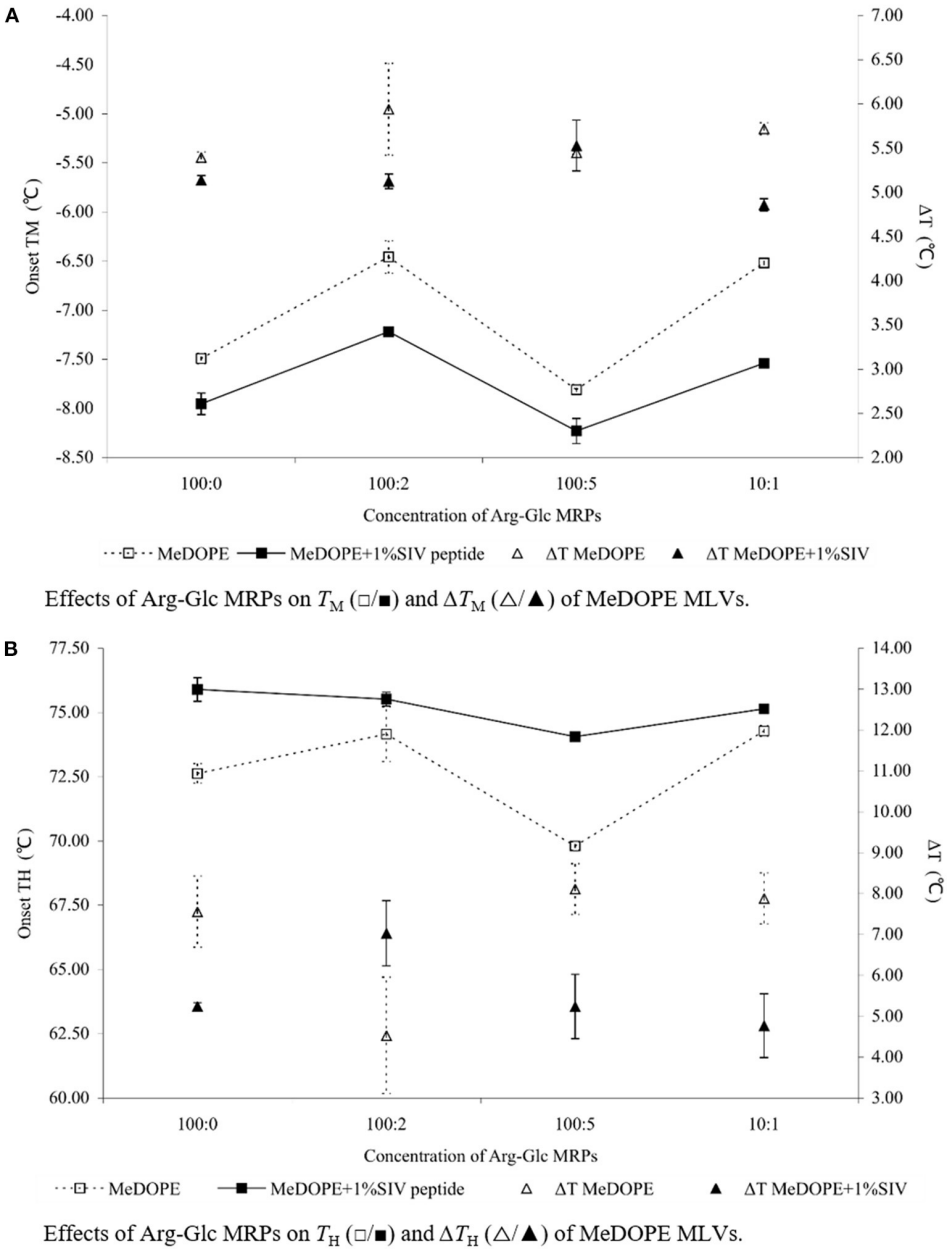
Effects of Arg-Glc MRPs on the phase transition behavior of MeDOPE MLVs obtained in the presence of 100:1 SIV peptide by DSC. Scan rate: 40 K/min. Scanning temperature range: −30–85°C. Lipid concentration: 100 mM. Data of 100:1 SIV on MeDOPE vesicles (labeled as “100:0”) was included for comparison (*n* = 4; *P* < 0.01).

In the *L*_α_-*H*_II_ phase transition (as shown in Figure 2B), the effects of Arg-Glc MRPs showed different impacts. The MRPs promoted the transition at 100:5 but inhibited the transition at the lower (100:2) or higher concentrations (10:1) in the absence of SIV. As shown in Figure 3, in the presence of 100:1 SIV, the effects of Arg-Glc MRPs became much milder. *T*_H_ of the MeDOPE vesicles was 72.6 ± 0.4°C, which was elevated to 75.9 ± 0.5°C in the presence of 100:1 peptide (*P* < 0.01) and brought back to 74.1 ± 0.1°C by adding 100:5 Arg-Glc MRPs (*P* < 0.01), and to 75.1 ± 0.1°C by adding 10:1 Arg-Glc MRPs (*P* < 0.01). Furthermore, the Δ*T*_H_ of *L*_α_-*H*_II_ phase transition was significantly affected by Arg-Glc MRPs, but only at 100:2. The opposite effects were observed in the presence or absence of SIV. The MRPs decreased the Δ*T*_H_ by 3°C in the absence of SIV, but increased the Δ*T*_H_ by 2.8°C in the presence of SIV (*P* < 0.01). At the higher concentrations of the MRPs, 100:5 and 100:10, the MRPs did not affect Δ*T*_H_ as potent as it was at 100:2.

### Structural Rearrangements in the Phase Transitions: SAXS Study

To gain more details of the structural rearrangements involved in the phase transition, small angle X-ray scattering (SAXS) was used to investigate the transitions from lamellar phase (*L*_α_) to inverted hexagonal phase (*H*_II_) and/or from *L*_α_ to inverted cubic phase (*Q*_II_) in the presence of Arg-Glc MRPs.

As shown in **Figure 5**, three phases, the lamellar, cubic, and hexagonal phase, were characterized with temperature-resolved SAXS in MeDOPE MLVs by determining the membrane structure-corresponding scattering density profiles at a heating rate of 1 K/min. At least two cubic structures, possibly the diamond and primitive bicontinuous phases, were observed while two orders of diffraction peaks in lamellar and hexagonal phase each were measured. The continuous existence and coexistence of different phases makes it possible to evaluate the influence of MRPs, SIV fusion peptide, and fusion inhibitor (LPC) on MeDOPE MLVs. For example, the *L*_α_-to-*H*_II_ and *L*_α_-to-*Q*_II_ phase transitions were promoted in the presence of Arg-Glc MRPs (100:5), indicated by the dropping *T*_H_ and *T*_Q_, as shown in **Figure 6**.

**Figure 4:**
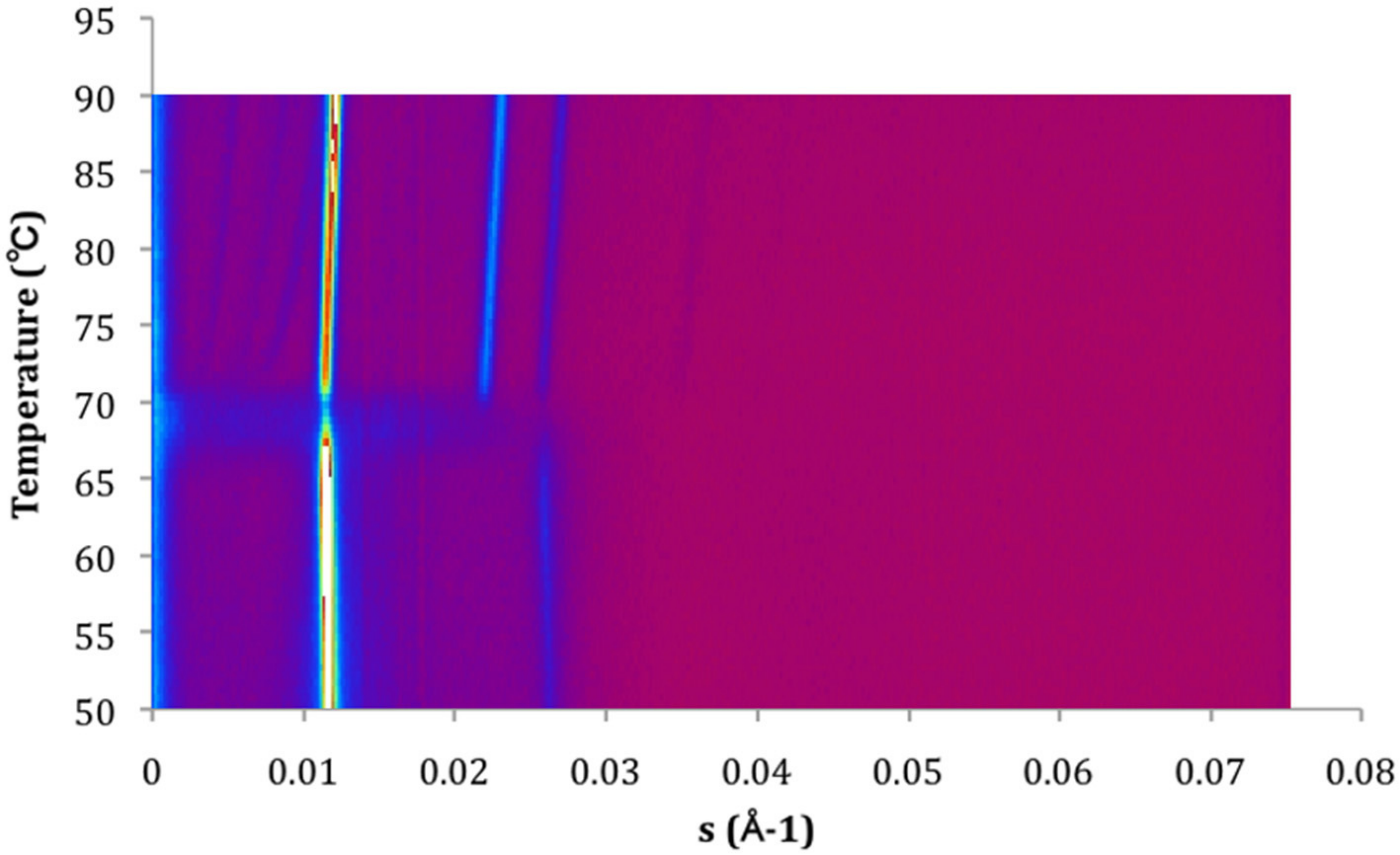
Contour plot of X-ray scattering of MeDOPE MLVs. Temperature scan rate of 1 K/min. The figure shows a *L*_α_-to-*H*_II_ transition around 70°C and a coexisting of *H*_II_ and *Q*_II_.

**Figure 5:**
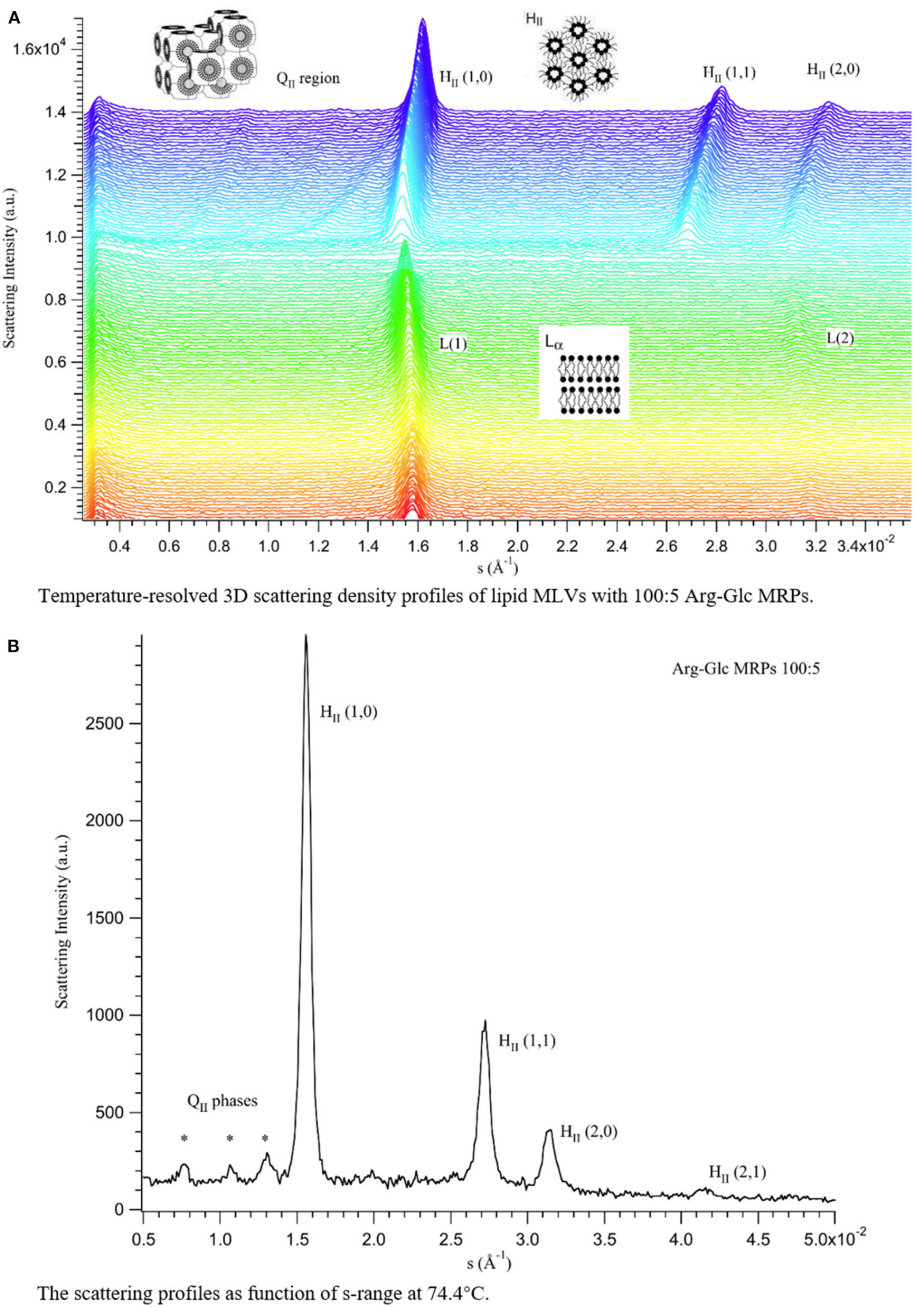
Scattering profiles of MeDOPE MLVs in the presence of Arg-Glc MRPs (100:5). **(A)** Temperature-resolved 3D scattering density profiles. **(B)** The scattering density profiles as a function of s-range at 74.4°C (*T*_H_ + 7.5°C). L(1) refers to the first-order scattering peak of *L*_α_ phase. L(2) refers to the second-order scattering peak of *L*_α_ phase. Q_II_ refers to the inverted cubic phase, in which three diffraction peaks were observed, labeled with ^*^. H_II_ (1,0)/(1,1)/(2,0)/(2,1) refer to the first and second order of the inverted hexagonal phase.

**Figure 6:**
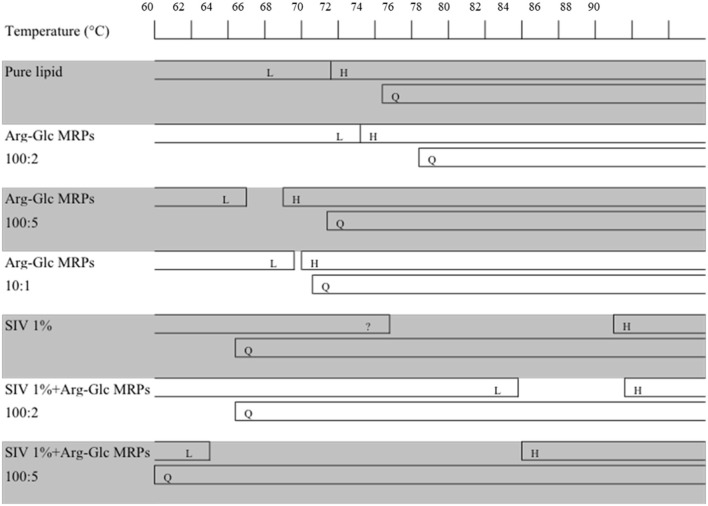
Schematic summary of the phase behavior of MeDOPE MLVs and the MeDOPE in the presence of Arg-Glc MRPs and/or SIV fusion peptide. The transition temperatures were measured by a temperature scanning at 1 K/min from 30 to 90°C with the temperature-resolved X-ray diffraction.

As shown in Figures 4, 5, the coexistence of *H*_II_ and *Q*_II_ phase was observed in the pure lipid vesicle. The *T*_H_ was 69.3°C. The inverted cubic phase started at 72.2°C. The scattering profile indicates that lipid bilayer in the cubic phase has more than one structure, possibly the double-diamond (*P*_n3m_) and primitive phase (*I*_m3m_). The addition of 100:2 Arg-Glc MRPs raised the *T*_H_ to 70.9°C and the *T*_Q_ to 74.3°C (Figure 6). However, when the concentration of Arg-Glc MRPs was increased to 100:5, the *T*_Q_ decreased to 69.4°C, while the *T*_H_ decreased to 66.9°C. At this proportion, the MRPs induced a 2°C gap between the *L*_α_ and *H*_II_ phase where no structure was observed. When the proportion of Arg-Glc MRPs reached 10:1, the *T*_H_ and *T*_Q_ were 1.3°C and 3.7°C lower than the blank vesicle, respectively.

SIV fusion peptide (1 mol%) lowered the *T*_Q_ to 64.3°C, but increased the *T*_H_ to 85.0°C. SIV weakened and broadened the scattering profile of liquid crystalline phase, and extended the end of this phase to 72.7°C (Figure 6, “SIV 1%”). However, since the shape and intensity of the Bragg peak were not identical to those of the normal *L*_α_ phase, a further analysis needs to be performed on the lattice spacing and electron density. Different from the pure lipid, SIV induced a coexistence of *L*_α_ and *Q*_II_ phase at the temperature ranging from 64.3 to 72.7°C, and a coexistence of *Q*_II_ and *H*_II_ phase at the temperature ranging from 85.0°C to the end of scan (90°C). This indicates the dominant impacts of SIV on the lipid phase behavior.

The low concentration of Arg-Glc MRPs (100:2) raised the *T*_H_ to 85.5°C in the lipid bilayer with 1% SIV, which was slightly higher than the *T*_H_ of SIV, whereas the *T*_Q_ was not affected. The end of the lamellar phase was raised by 6.9°C, which narrowed down the gap from *L*_α_ to *H*_II_. The rise in the *T*_H_ and the extension of lamellar phase both suggest a stabilizing effect of Arg-Glc MRPs (100:2) on SIV containing lipid bilayers. The *T*_Q_ remains the same as that of SIV, which implies that the cubic phase is more preferable than the hexagonal phase in the presence of the MRPs.

At medium concentration, Arg-Glc MRPs lowered all of the *T*_H_, *T*_Q_, and the end of the *L*_α_ phase in the presence of SIV while inducing the largest temperature gap between the *L*_α_ and *H*_II_ phase (Figure 6). This indicates a destabilizing effect of MRPs on the lamellar structure. The stronger scattering intensity of all the phases observed upon 100:5 MRPs may suggest the more ordered packing of lipids in each phase.

## Discussion

The polyphasic structural transformation of MeDOPE has been extensively studied with a variety of techniques (Ellens et al., 1986; Siegel et al., 1994; Colotto et al., 1996; Harroun et al., 2003; Kusube et al., 2006). The cubic structure is observed as a metastable phase when the vesicles are transferring from the lamellar phase to the hexagonal phase. This depends on the heating rate (thermal scan rate) across the lamellar-to-hexagonal phase transition (van Gorkom et al., 1992).

A wide range of temperature has been reported for the inverse hexagonal phase transition. As revealed in an X-ray diffraction study reported by Cherezov, the *Q*_II_ phase appears at 59.1°C while the *H*_II_ phase appears at 63.5°C, at a scan rate of 1.5 K/h. *T*_H_ increased by 2°C when the scan rate was raised to 6 K/h (Cherezov et al., 2003). Harroun et al. (2003) reported the *T*_H_ of 64°C and the *T*_Q_ of ~75°C at the scan rate of 30 K/h. In this study, there is a main phase transition at 72.6°C recorded by DSC at the much faster scan rate, 40 K/min. The transition is presumably *T*_H_, that is from the lamellar phase (*L*_α_) to the inverted hexagonal phase (*H*_II_), since the hexagonal phase is the major visible structure of MeDOPE MLVs at high temperature. The *T*_Q_ between the lamellar phase (*L*_α_) and the inverted cubic phase (*Q*_II_) was about 62.0°C. The *T*_H_ determined in this study is much higher than the reported value. A remarkable difference among these measurements is the scan rate. It is 40 K/min in the DSC measurement compared to 1.5–30 K/h in the X-ray diffraction measurements. There was another phase transition at the lower temperature (~62°C), presumably the *T*_Q_, which did not appear in every single isothermal scan of DSC measurement. Data are given in Figure 1B for reference.

Firstly, either stabilization or destabilization effects of Arg-Glc MRPs were observed on the different phase of the MLVs. At the concentration of 100:5, Arg-Glc MRPs destabilized the MeDOPE bilayers in the lamellar phase, both gel and liquid crystalline phases, and accordingly lowered the *T*_M_ and *T*_H_ (Figures 1A,C). However, when the ratio went up to 10:1, the MRPs stabilized the bilayer in the lamellar phase and elevated the *T*_M_, *T*_Q_, and *T*_H_. At ratios higher than 10:1, the MRPs destabilized the bilayers again. However, *T*_H_ was constantly going down, like what occurred to *T*_M_ and *T*_Q_. *T*_H_ rose back to about the same temperature as that of pure MeDOPE, at a ratio of 10:4, indicating another stabilization in the lipid structure before it transfers to inverted hexagonal phase. At the highest ratio, a bilayer breaking down was implied by the acute drop in *T*_H_ and rise in Δ*H*_f_.

Taking together the effects of Arg-Glc MRPs across all three phase transitions, a general pattern of MRPs' effects emerges. The stabilization actions of the MRPs are split into two stages. Firstly, the MRPs fill up the spaces in the head group region of leaflet and keep the hydrophilic heads away from each other. This prevents the development of negative curvature strain in the bilayers and subsequently inhibits the lamellar/non-lamellar transition. The bilayers prefer to remain in gel or liquid crystal phase. MRPs at a molar ratio of 10:1 performed the strongest stabilization effects of this kind. Secondly, when more MRP molecules accumulate in the bilayers, they start to destabilize the lamellar structure of lipids and favor the lamellar-inverted cubic transition. Once the lipids transfer to non-lamellar structure, the lipids prefer to stay in the cubic phase rather than the hexagonal phase. The best example for this kind is bilayers containing Arg-Glc MRPs at a molar ratio of 10:4. MRPs decreased the *T*_M_ and *T*_Q_ but raised the *T*_H_. At concentrations beyond this point, the bilayer was further destabilized and became easier to transfer to the inverted hexagonal phase.

A drop in transition temperature can correlate with a rise in heat consumption, such as that observed in MeDOPE MLVs in the presence of Arg-Glc MRPs. For example, Arg-Glc MRPs, at the molar ratio of 10:5, decreased the temperature of lamellar-inverted hexagonal transition and increased the heat consumption. Similar outputs were observed in the gel-liquid crystalline phase transition at higher molar ratios than 10:1. This implies that Arg-Glc MPRs destabilize the lipid bilayers at high concentrations and promote the phase transition from an ordered to a less ordered status. Meanwhile, due to the possible coexistence of several sub-phases induced by interference of MRPs, the amount of energy in terms of heat as a function of time, was increased to overcome the overall energy barrier and encourage the formation of a regular structure.

Secondly, the SIV fusion peptide changed the phase behavior of the lipid vesicles, which was regulated by Arg-Glc MRPs. In the DSC study, SIV fusion peptide raises the *T*_H_ of MeDOPE by 4°C but decreases *T*_M_ at a high temperature scan rate (40 K/min). This is consistent with X-ray diffraction data (Harroun et al., 2003), in which the SIV peptide (lipid/peptide = 100:1) raises *T*_H_ by 10°C (to about 74°C) at the temperature scan rate of 30 K/h, while at 100:2 SIV, the hexagonal phase is eliminated. In contrast, the SIV peptide (lipid/peptide = 100:1) raises *T*_H_ to about 76°C at the scan rate of 40 K/min in this study. At 100:2 SIV, the hexagonal phase is still observed as *T*_H_ is about 77°C. It has been described for *T*_H_ to be scan-rate-dependent (Colotto et al., 1996), which is presumably varied along the different temperature scan rates. This might explain why the *T*_H_ of MeDOPE MLVs obtained in my DSC study is higher by 2°C than that of X-ray data at 60°C/h. However, one can also attribute this variation to the differences in the sensitivity of the two methods. By analyzing the Gaussian peaks, a minor change in structure of bilayers can be detected and examined with X-ray scattering. DSC is capable of examining main phase transition events like lamellar-to-hexagonal phase transition but has a limited capacity to track transient structures of intermediates, e.g., cubic phase, at the relatively high scan rate employed in this study.

SIV promotes the breakdown of lamellar structure and the formation of the cubic phase (Harroun et al., 2003). Some effects of SIV on lamellar-to-cubic phase transition, such as lowering the *T*_Q_, are not readily measured by DSC as shown in Figure 1B, since the transient phases are in continuous change. However, the fusogenic nature of the peptide is still implied by the drop in Δ*T*_H_ and heat consumption of the *L*_α_-*H*_II_ phase transition. The 2°C decline in the temperature range of this transition, in the presence of 100:1 SIV, possibly suggests that a relatively uniform lipid packing is installed in the transient structure prior to the occurrence of the *L*_α_-*H*_II_ transition. Furthermore, in terms of its effect on *T*_M_, *T*_H_, Δ*H*_f_, and Δ*T*_H_, the 100:2 SIV peptide is more potent than 100:1 SIV, but not much. It is also consistent with previous X-ray diffraction data reported by Harroun et al.

Arg-Glc MRPs partly offset the effects of SIV peptide on MeDOPE phase transition, in a concentration-dependent manner. In the presence of 100:1 SIV, the MRPs at 100:2 and 10:1 stabilize the gel phase when 100:5 MRPs destabilize the gel phase and liquid crystal phase. The MRPs reversed the destabilizing effects of SIV fusion peptide on the lamellar phase of liposome, to potentially inhibit membrane fusion induced by SIV.

By measuring the phase transition as a function of temperature with SAXS, the influences of Arg-Glc MRPs on the SIV peptide-induced membrane fusion are revealed. The rise in the transition temperature of non-lamellar phases, induced by 100:2 Arg-Glc MRPs, implies a stabilization of lamellar structure conducted by the MRPs. However, the effects of Arg-Glc MRPs on *T*_H_ and *T*_Q_ are reversed by increasing their proportion in lipid bilayer to 100:5. The MRPs destabilize the lamellar bilayer and encourage the formation of non-lamellar phase. At a higher MRP concentration (10:1), the promoting effects of Arg-Glc MRPs on the inverted hexagonal phase become weaker, while the *T*_Q_ was dropping. This implies that the high concentration of Arg-Glc MRPs mildly affected *L*_α_-to-*H*_II_ transition while promoting *L*_α_-to-*Q*_II_ transition. This observation is in good agreement with the phase transition studies conducted with DSC.

The action of fusion peptides on the inverse hexagonal phase is separated from their ability to inducing inverse cubic phase (Darkes et al., 2002). It has been reported that the SIV peptide dramatically delayed the inverse hexagonal phase while inducing “a gap between the phase where no structure was presented” (Harroun et al., 2003). In my study, SIV acts very similarly except the gap between *L*_α_ and *H*_II_ phases is covered up by the extension of a lamellar-like phase. SIV tends to promote the formation of an inverse cubic phase when bypassing the intermediate structures leading to the hexagonal phase. Arg-Glc MRPs (100:2) stabilize the bilayer and increase the temperature range of lamellar phase by about 7°C. However, the MRPs at 100:5 destabilize the lamellar phase and promote the formation of the inverse cubic phase and inverse hexagonal phase, while partially offsetting the delay in lamellar-to-hexagonal transition caused by the fusion peptide. Therefore, the overall influences of MRPs at 100:5 on the peptide-induced membrane fusion remains uncertain and warrants further study.

## Conclusion

In summary, Arg-Glc MRPs stabilize the lamellar phase and inhibit the SIV-induced negative curvature strain of bilayer at a low concentration (100:2). Although this inhibitory effect seemed to fade away at higher concentrations (100:5) of MRPs, the regulating activity of the MRPs upon lipid lamellar-to-non-lamellar phase transition indicates their potential role in modulating the membrane-related biological events, e.g., viral membrane fusion. The real antiviral efficacy of Arg-Glc MRPs, based on this novel mechanism, is worth further evaluation by using living biological models *in vitro* and *in vivo*.

## Data Availability Statement

The original contributions presented in the study are included in the article/supplementary materials, further inquiries can be directed to the corresponding authors.

## Author Contributions

LK planned and conducted the experiments and wrote the manuscript. PR and JB participated in the data analysis and editing the manuscript. FS helped with the sample preparations and SAXS experiments. MR helped with SAXS experiments and the data analysis as the synchrotron station scientist. JZ helped with chemical tests and writing up. All authors contributed to the article and approved the submitted version.

## Conflict of Interest

The authors declare that the research was conducted in the absence of any commercial or financial relationships that could be construed as a potential conflict of interest.
